# Screening for *in vitro* phototoxic activity of methanol extracts of *Croton campestris* A., *Ocimum gravissimum* L. & *Cordia verbenaceae* DC

**DOI:** 10.4103/0971-5916.73392

**Published:** 2010-11

**Authors:** Edinardo F.F. Matias, Karla K.A. Santos, José Galberto M. Costa, Henrique D.M. Coutinho

**Affiliations:** * Laboratory of Microbiology & Molecular Biology, University of the Region of Cariri Crato (CE), Brazil; ** Laboratory of Research on Natural Products, University of the Region of Cariri, Crato (CE), Brazil

Sir,

Medicinal plants have been the subject of intense research due to their potential as sources of commercial drugs or as lead compounds in drug development^1^,^2^. Various aspects of the photochemistry and photobiology of natural products, including their potential as therapeutic agents, have been reviewed^3^–^7^.

An increasing number of natural products from plants have been shown to exhibit light-mediated biological activity against viruses, microorganisms, cells and insects^8^. Although there is a great amount of published data regarding the antimicrobial properties of medicinal plants, there is no information on light-activated biological activities from this natural resource, that could represents an interesting source of photosentizers^9^,^10^.

The species *Croton campestris* is a shrub endemic to Brazil, and is used to combat various diseases^11^. *Ocimum gratissimum L*. is an aromatic subshrub from Asia and Africa^12^. The essential oil from the leaves of this species has shown antimicrobial activity against various microorganisms such as *Staphylococcus aureus, Bacillus* spp, *Pseudomonas aeruginosae, Klebisiella pneumoniae, Proteus mirabilis* and *Leishmania amazonensis*^12^–^14^. The species of the genus *Cordia* are found in all tropical and subtropical regions^15^. The crude extract of the aerial part is utilized by indigenous people against inflammatory processes with topical use. It has been suggested that the flavonoid artemetin is the compound responsible for the anti-inflammatory activity of this species^16^.

Many medicinal plants contain substances that can initiate adverse reactions, requiring a rigorous control of the quality of cultivation, collection of the plants, extraction of their constituents, and in the preparation of the final product^17^. Focusing on the concept of photochemistry and photochemotherapy, we examined three potentially useful Brazilian medicinal plants and found their extracts to show light-mediated antimicrobial activities.

The present investigation was a preliminary screening for phototoxic activity in natural products with antibacterial activities from medicinal plants used in Brazil. The extracts of *Croton campestris A., Ocimum gravissimum L*. and *Cordia verbenaceae DC*. were tested as sources of phototoxic compounds.

The bacterial strains used were: *Escherichia coli* (ATCC 8538) and *Staphylococcus aureus* (ATCC 6538). All strains were maintained on heart infusion agar slants (HIA, Difco Laboratories Ltd., USA) and prior to assay, the cells were grown overnight at 37°C in brain heart infusion (BHI, Difco Laboratories Ltd., USA). 8-Methoxy psoralen (8-MOP) (Sigma Chemical Co., USA) was dissolved in sterile water. Disks with norfloxacin were obtained from Laborclin, Brazil. Leaves of *C. campestris A., O. gravissimum L*. and *C. verbenaceae DC*. were collected in the county of Crato, Ceará State, Brazil. The plant material was identified and a voucher specimen was deposited with the respective identification numbers (Table I).

**Table I:** Botanical families, species and voucher number of the plants used in this study

Family	Species	Abbreviation	Number	Herbarium
Euphorbiaceae	*Croton campestris*	MECC	#7095	UFRN
Boraginaceae	*Cordia verbenacea*	MECV	#044171	Prisco Bezerra-UFC
Lamiaceae	*Ocimum gratissimum*	MEOG	#3978	Dárdano Andrade Lima-URCA

Amounts of 31.2, 58.82 and 31.4 g of leaves from *C. campestris, O. Gratissimum* and *C. verbenacea*, respectively, were dried at room temperature and powdered. The material was extracted by maceration using 1 L of methanol as solvent at room temperature, and the homogenate was allowed to stand for 72 h at room temperature. The extracts were then filtered and concentrated under vacuum in a rotary evaporator (model Q-344B – Quimis, Brazil) using a warm-water bath (model Q-214M2 - Quimis, Brazil). Each plant material yielded 1.74, 2.49 and 1.74 g of extract, respectively. For the tests, the dry extract material was dissolved in DMSO (10 mg/ml).

Assays were performed according to Lopez *et al*^4^. As positive controls, a disk of norfloxacin was used as a standard antibiotic for bacteria with photoactivated properties. 8-MOP (10 mg/ml) in water was utilized as a positive control requiring light for activation. Twenty microlitres of each extract were added to blank disks with 5 mm of diameter. These disks were placed on the surface of the medium inoculated by spread plate method with bacteria. To monitor for light-activated antimicrobial activities, two replicate experiments were carried out. One replicate plate was exposed to ultraviolet (UV) light (5 W/m^2^, 320–400 nm from four Sylvania F20T12-BLB lamps, maximum emission at 350 nm) for 2 h, while the other was kept in the dark. The plates were incubated at 37°C overnight; the inhibition zones were determined and recorded (Table II).

**Table II:** Diameter of Inhibition zones (mm) derived of light-mediated antimicrobial activity of methanol extracts from *Croton campestris, Ocimum gratissimum* and *Cordia verbenaceae*

	SA6538			EC8539		
	UV–	UV+	Enhancement (%)	UV–	UV+	Enhancement (%)
MECC	5 ± 0	8 ± 0	60	5 ± 0	5 ± 0	0
MEOG	5 ± 0	12 ± 2	140	5 ± 0	5 ± 0	0
MECV	5 ± 0	12 ± 0	140	5 ± 0	5 ± 0	0
NOR^a^	26 ± 1	35.5 ± 4.5	36,5	14,5 ± 2	34 ± 5	137.9
8MOP^b^	7.5 ± 0.5	26 ± 2	246,7	5 ± 0	18 ± 0	260

Values are mean ± SD of three observations MECC, methanol extract of *Croton campestris*; MEOG, methanol extract of *Ocimum gratissimum*; MECV, methanol extract of *Cordia verbenacea*; UV, without UV irradiation; UV+, with UV irradiation; –, no inhibition;

a Norfloxacin (10 μg/disk): positive standard;

b 8-Methoxyl-psoralen (10 mg/ml); SD, standard deviation

A substantial phototoxic effect was seen with the three extracts against the *S. aureus* but not against the *E. coli* strain. Methanol extract of *O. gratissimum* (MEOG) and *C. verbenaceae* (MECV) were the extracts that showed the highest phototoxic activity. Besides the enhancement of the light mediated toxic activity of the extracts, these activities were lower than that observed by the norfloxacin and 8-MOP (Table II). Our results indicated the phototoxic potential of these extracts and highlight the necessity of more studies to evaluate the possible applications of these natural products.

Many plant substances when exposed to UV or visible light exhibit phototoxicity and are referred to as phototoxins or photosensitizers^18^. A large variety of plants and fungi of various families possess phototoxic substances, possibly serving as natural defense agents against insects and nematodes or against predation or herbivory^8^,^19^. This activity is due to two mechanisms: an indirect effect, through the production of free radicals, or a direct effect, such as with furocumarins which interact with DNA^20^.

Some compounds with phototoxic activity have been shown to have biological activity when photoactivated. Hypericin and hypocrelin, isolated from *Hypericum perforatum* and *Hypocrella bambuase*, respectively, have shown anti-HIV and antitumour activity^8^, while other studies demonstrated antibacterial activity with UV light activation^20^.

The results of the present study show that light can be utilized for antimicrobial activity of phytoconstituents obtained from methanol extracts of the *species C. campestris, O. gratissimum* and *C. verbenaceae*, suggesting that phytochemical investigations are necessary to determine if these plants could serve as a source of natural products with phototoxic activities, which could be an interesting and alternative source of natural compounds to be used in the treatment of skin disorders and bacterial infections.
